# Soybean *Bradyrhizobium* spp. Spontaneously Produce Abundant and Diverse Temperate Phages in Culture

**DOI:** 10.3390/v16111750

**Published:** 2024-11-07

**Authors:** Vanessa A. Richards, Barbra D. Ferrell, Shawn W. Polson, K. Eric Wommack, Jeffry J. Fuhrmann

**Affiliations:** 1 Department of Biological Sciences, University of Delaware, Newark, DE 19716, USA; 2 Department of Plant and Soil Sciences, University of Delaware, Newark, DE 19716, USA; 3 Department of Computer and Information Sciences, University of Delaware, Newark, DE 19713, USA; 4 Microbiology Graduate Program, University of Delaware, Newark, DE 19713, USA

**Keywords:** *Bradyrhizobium*, soybean, bacteriophages, phage induction, lysogeny

## Abstract

Soybean bradyrhizobia (*Bradyrhizobium* spp.) are symbiotic root-nodulating bacteria that fix atmospheric nitrogen for the host plant. The University of Delaware *Bradyrhizobium* Culture Collection (UDBCC; 353 accessions) was created to study the diversity and ecology of soybean bradyrhizobia. Some UDBCC accessions produce temperate (lysogenic) bacteriophages spontaneously under routine culture conditions without chemical or other apparent inducing agents. Spontaneous phage production may promote horizontal gene transfer and shape bacterial genomes and associated phenotypes. A diverse subset (n = 98) of the UDBCC was examined for spontaneously produced virus-like particles (VLPs) using epifluorescent microscopy, with a majority (69%) producing detectable VLPs (>1 × 10^7^ mL^−1^) in laboratory culture. Phages from the higher-producing accessions (>2.0 × 10^8^ VLP mL^−1^; n = 44) were examined using transmission electron microscopy. Diverse morphologies were observed, including various tail types and lengths, capsid sizes and shapes, and the presence of collars or baseplates. In many instances, putative extracellular vesicles of a size similar to virions were also observed. Three of the four species examined (*B. japonicum*, *B. elkanii*, and *B. diazoefficiens*) produced apparently tailless phages. All species except *B. ottawaense* also produced siphovirus-like phages, while all but *B. diazoefficiens* additionally produced podovirus-like phages. Myovirus-like phages were restricted to *B. japonicum* and *B. elkanii.* At least three strains were polylysogens, producing up to three distinct morphotypes. These observations suggest spontaneously produced phages may play a significant role in the ecology and evolution of soybean bradyrhizobia.

## 1. Introduction

The demand for food crops is rapidly increasing with the world’s population, which is estimated to reach 9.8 billion by 2050 [1]. Soybean (*Glycine max*) is an important protein crop and source of edible and industrial oil. In the United States, soybean accounts for 33% of the total cultivated land. *Bradyrhizobium* spp. are soil bacteria that nodulate soybean roots and fix atmospheric N_2_ into NH_3_ for the plant host, reducing the need for environmentally harmful fertilizers. Symbiotically superior strains of these bacteria can increase crop yield by 32% when compared with indigenous soil strains [2] without the use of synthetic fertilizers, which contribute to air and water pollution. However, bradyrhizobia strains differ in their abilities to nodulate and fix nitrogen. Nodulation by less efficient strains can lead to lower soybean yield. The University of Delaware *Bradyrhizobium* Culture Collection (UDBCC) was created to study the diversity and ecology of bradyrhizobia and consists of 340 Delaware soybean root nodule isolates and 13 USDA reference strains [3]. Our earlier studies [4] revealed that some UDBCC strains produce temperate phages spontaneously in routine culture, i.e., without apparent external inducing agents.

Temperate phages integrate their genome into a host genome where they reside as a prophage, a condition known as lysogeny. More rarely, prophages can exist as extrachromosomal elements [5]. Prophages replicate indefinitely as the host cell divides but may later excise from the host genome and enter a lytic cycle to produce free virions, a process called induction. Spontaneous phage production, a phenomenon in which prophage induction and subsequent viral replication and virion release occur without apparent exogenous inducing agents, was first described in the early 1950s [6] and sporadically since for various bacterial taxa (e.g., [7,8,9,10]). More recently, Joglekar et al. [4] described high levels of spontaneous phage production in three strains of soybean bradyrhizobia and conclusively demonstrated that virions produced arose from prophages rather than satellite phages or free phages endemic within the culture media. Nanda et al. [11] reviewed the possible mechanisms of spontaneous phage production, especially virion production caused by stochastic reductions in the levels of induction suppressor molecules in a subpopulation of cells in a culture.

Prophage induction can be triggered by the host SOS response, which in turn is activated by damage to the host cell DNA [12]. Thus, induction provides a way for a prophage to escape from a physiologically challenged or dying host. Induction can result from environmental stressors such as elevated temperature, UV radiation, toxins, and osmotic shifts, some of which may stall bacterial DNA replication and activate the SOS response system. Conversely, SOS-independent induction has also been reported [13], for example, via quorum sensing events [14,15].

Lysogenic infection in bradyrhizobia was first reported for *B. japonicum* serogroup 123 strain L4-4 [16]. Marsh and Wellington [17] theorized that lysogenic relationships are favored in the soil environment due to reduced close contact and lowered metabolic activity of microbes, resulting in reduced replication opportunities for free viruses. Prophages can provide their hosts (lysogens) a selective advantage over their non-lysogenic counterparts [18,19]. These benefits include possible super-infection immunity against other viruses and the acquisition of useful traits by horizontal gene transfer (HGT) from previous hosts, i.e., via generalized or specific transduction events. Notably, Joglekar et al. [4] used a read-mapping strategy to accurately delineate prophages in bradyrhizobia genomes and found compelling evidence of specialized transduction in the associated spontaneously produced phages.

Little information exists on the biotic constraints affecting rhizobial microsymbionts [20] compared with more well-studied abiotic factors. With respect to soybean bradyrhizobia in particular, virulent (lytic) viruses were shown to reduce susceptible populations of rhizobia and, therefore, their ability to nodulate and biologically fix nitrogen for the host plant [21,22]. However, little is known about the effects of temperate phage infection in soybean bradyrhizobia. Given that phages in soil are estimated to outnumber bacteria by 5- to 1000-fold [23] and approach 10^10^ phages g^−1^ soil in certain biomes [24], it stands to reason that bradyrhizobia populations are commonly infected and altered by both virulent and temperate phages and that these interactions warrant study to elucidate bradyrhizobia diversity and ecology and ultimately enhance the soybean–*Bradyrhizobium* symbiosis.

In this study, epifluorescence microscopy was used to screen representative accessions from the UDBCC for spontaneous production of virus-like particles (VLPs) in routine laboratory culture. Epifluorescence microscopy has been the “gold standard” for virus enumeration due to its relatively low costs and high throughput [25]. In the past, phage enumeration by transmission electron microscopy (TEM) was common [26,27], but this method is error-prone and involves both lengthy preparation and the use of an electron microscope, which may not be readily accessible. However, TEM was used in this study to document virion morphology [28,29]. The quantification of spontaneous phage production and morphological features serve as initial steps toward understanding rhizobiophage diversity and potential effects on the *Bradyrhizobium*–soybean symbiosis.

## 2. Materials and Methods

### 2.1. Bacterial Strains and Cell Growth

A subset of 98 diverse strains from the UDBCC, representing both root nodule isolates sampled from 29 Delaware soybean farms (n = 85) and USDA reference strains (n = 13), was used in this study, as described by Joglekar et al. [3]. Criteria for selecting the subset included contrasting serological reaction, cellular fatty acid composition, and Internal Transcribed Spacer-Restriction Fragment Length Polymorphism (ITS-RFLP) grouping. Reference strains obtained from United States Department of Agriculture *Rhizobium* Culture Collection (Beltsville, MD, USA) are denoted by the prefix “USDA”, whereas the naming convention for Delaware field isolates is first by county of sampling (K, N, or S for Kent, New Castle, and Sussex counties, respectively) followed by farm number within a given county (e.g., 01), and lastly, an isolate code (A–L) for a given farm (typically, 12 root nodule isolates were obtained from each farm). Bradyrhizobia cultures were retrieved from 25% glycerol stocks (25% glycerol (Fisher Scientific, Waltham, MA, USA), 75% Modified Arabinose Gluconate (MAG); ATCC medium 2233) stored at −80 °C. Strains were individually grown in MAG broth for 7 to 10 days (late-log to early-stationary phase) at 28 °C with shaking at 155 rpm prior to characterization.

### 2.2. Spontaneous Induction and Enumeration of Phage

Aliquots (1.0 mL) of bacterial cultures were centrifuged for 15 min at 10,000 RCF to pellet cells, and a 500 µL aliquot of the resulting supernatants was filtered through 0.2 µm syringe filters (Whatman^®^; Maidstone, UK). The filtrates (250 µL) were collected on 0.02 µm Whatman^®^ Anodisc membrane filters and stained with 200 µL of 2× (final concentration) SYBR Gold DNA stain (Biorad, Hercules, CA, USA) for 15 min and washed twice with 500 µL of 0.02 µm filtered SM buffer (50 mM Tris–HCl, pH 7.5, 100 mM NaCl, 10 mM MgSO_4_, and 0.01% gelatin; Fisher Scientific). The Anodisc filter was transferred to a microscope slide, and 30 µL of antifade solution (0.1% *p*-phenylenediamine; Sigma-Aldrich, St. Louis, MO, USA) was applied to suppress photobleaching, followed by a cover slip. Virus-like particles (VLPs) retained by the Anodisc filter were digitally imaged using an epifluorescence microscope equipped with a fluorescein (FITC) filter set at 1000× magnification. For each micrograph, image processing software (Serif PhotoPlus X8 v18.0.0.15) was used to superimpose a virtual grid with squares of known area, and VLPs in ten randomly selected squares were counted and averaged to estimate VLP abundance. Recorded counts for each strain were converted to number of VLPs mL^−1^ of original culture.

### 2.3. Electron Microscopy of Phages

Strains showing VLP production greater than 2.0 × 10^8^ VLPs mL^−1^ (n = 44) were prepared for morphological examination by transmission electron microscopy (TEM). Cultures (15 mL) grown as previously described were centrifuged at 1000 RCF for 30 min to pellet bacterial cells. Supernatants containing viruses were concentrated using 100 kDa Amicon^®^ Ultra-15 centrifugation filter units (Millipore, Lexington, MA, USA) at 5000 RCF for 25 min. The concentrated phages in the retentate were imaged at the University of Delaware Bio-Imaging Center (Newark, DE, USA). Copper grids (400 mesh) with a carbon film (Electron Microscopy Sciences, Hatfield, PA, USA) were glow discharged using a Pelco easiGlow™ glow discharge cleaning system (Ted Pella, Inc., Redding, CA, USA), rendering the film hydrophilic. The grids were floated on a drop of phage concentrate for several seconds, washed on three drops of water, and then negatively stained with 2% uranyl acetate (aq) (Fisher Scientific). Upon drying, samples were examined at 31,500× or 20,000× magnification using a Libra 120 transmission electron microscope (Zeiss, Oberkochen, Germany) operating at 120 kV, and images were acquired with an Ultrascan 1000 CCD (Gatan, Warrington, PA, USA). ImageJ Fiji 2.0.0 [30] (National Institute of Health, Bethesda, MD, USA) was used to measure the capsid, collar, tail, and baseplate dimensions. Capsid volumes were calculated using the formula V = 4/3πa^2^c, where V = volume of an ellipsoid, a = semi-axis of the capsid width (i.e., capsid width/2), and c = semi-axis of the capsid length (i.e., capsid length/2) [31].

### 2.4. Analysis and Statistics

A phylogenetic tree based on host strain Internal Transcribed Spacer (ITS) region DNA sequence diversity was used to explore possible relationships between VLP production and genetic differences among the strains [3]. An ITS tree was constructed in Geneious v10.2.6 (https://www.geneious.com, accessed on 2 November 2024; GraphPad Software, LLC, Boston, MA, USA) using MAFFT (Multiple Alignment using Fast Fourier Transform) [32] (Geneious plug-in, v1.4.0) and FastTree [33] (Geneious plug-in, v2.1.12) and visualized with Iroki [34]. ITS and 16S rRNA gene sequence data are available under NCBI BioProject PRJNA36297.

Capsid diameter, capsid volume, and tail length were grouped by phage morphotypes to document the distribution and variability of the various morphological measurements among the four *Bradyrhizobium* host species. Boxplots were made using the ggboxplot package in R v4.2.1.

## 3. Results

It is important to note that two independent measures were used in the observation of phages produced by the bradyrhizobial cultures. The first was epifluorescence microscopy, which was capable of identifying virus-sized nucleic acid-containing particles (most likely containing dsDNA), here referred to as virus-like particles (VLPs). Epifluorescence microscopy for counting VLPs was applied to a representative subset of 98 phenotypically and phylogenetically diverse soybean bradyrhizobia accessions from the UDBCC. The second was direct TEM observation of culture supernatants of 44 of the 98 accessions that produced relatively greater VLP counts to identify particles based on morphology. Transmission electron microscopy observations provided the strongest evidence for spontaneous phage production.

### 3.1. Spontaneous Production of VLPs

**Figure 1:**
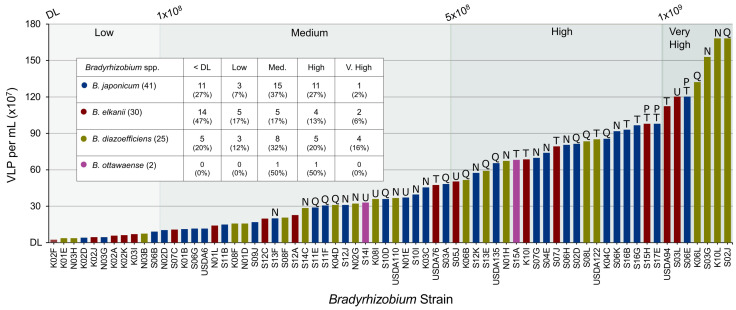
Numbers of virus-like particles (VLPs) spontaneously produced under routine laboratory culture of soybean *Bradyrhizobium* spp. Bars are colored according to species: *B. japonicum* (blue), *B. elkanii* (red), *B. diazoefficiens* (green), and *B. ottawaense* (purple). VLP production category ranges are shown in the inset. The detection limit (DL) was 1 × 10^7^ VLP mL^−1^. The table shows the number and percentage of each VLP production category for a given species. Letter codes above the bar for a given strain indicate the virion morphological category observed from TEM images: T = tailed virions, N = tailless virions with clearly icosahedral capsids, Q = tailless virions possibly observed but with questionable icosahedral morphology, U = unconfirmed presence of virions (no virions apparent in TEM images), P = polylysogenic strain; strains without a letter code were not examined for virion production by TEM.

UDBCC accessions (n = 98) examined by epifluorescent microscopy, and previously identified through ITS and 16S rDNA amplicon sequencing [3], were *Bradyrhizobium japonicum* (n = 41; sometimes denoted in this report by the suffix “-Bj” to the isolate or strain identifier), *B. diazoefficiens* (n = 25; “-Bd”), *B. elkanii* (n = 30; “-Be”), and *B. ottawaense* (n = 2; “-Bo”). Sixty-eight of the 98 strains (69.4%) produced detectable (>10^7^ mL^−1^) VLPs spontaneously (Figure 1). VLPs were produced in culture without any apparent inducing agents. VLP counts were arbitrarily binned into five categories: Very High (>1 × 10^9^ VLP mL^−1^; n = 7 strains), High (5 × 10^8^ to 1 × 10^9^ VLP mL^−1^; n = 21), Medium (1 × 10^8^ to 5 × 10^8^ VLP mL^−1^; n = 29), Low (1 × 10^7^ to 1 × 10^8^ VLP mL^−1^; n = 11), and Not Detectable (below detection limit of 1 × 10^7^ VLP mL^−1^; n = 30). The four strains producing the greatest numbers of VLPs (1.32 × 10^9^ to 1.68 × 10^9^ VLP mL^−1^) were in the species *B. diazoefficiens* and were isolated from four geographically separated (10 to 70 km distant) farms in Delaware (Figure 1). Also assigned to the Very High category were one *B. japonicum* and two *B. elkanii* strains, the latter including reference strain USDA94. All three of the dominant species of *Bradyrhizobium* in the UDBCC (*B. japonicum*, *B. elkanii*, and *B. diazoefficiens*) were represented in each of the five categories of VLP production. The species *B. elkanii* had a notably greater percentage of strains (47%) producing no detectable VLPs (<1 × 10^7^ VLP mL^−1^) compared with *B. diazoefficiens* (20%) and *B. japonicum* (27%).

**Figure 2:**
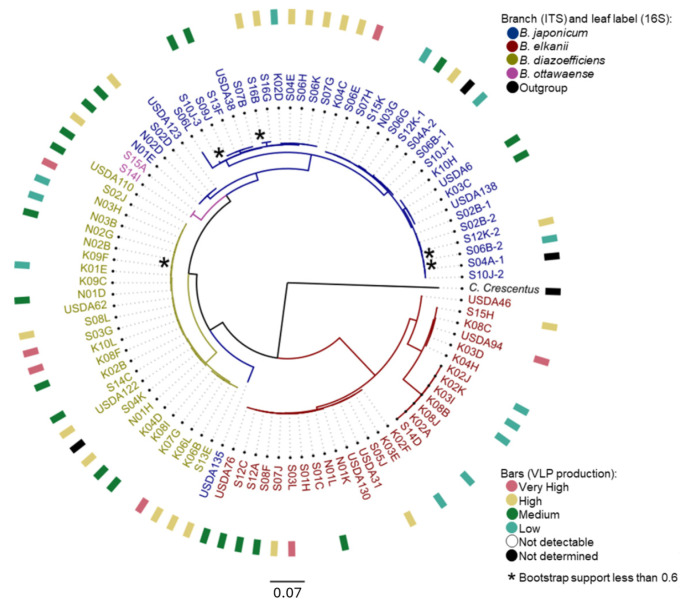
Phylogenetic tree of the internal transcribed spacer (ITS) region of soybean *Bradyrhizobium* spp. with associated levels of spontaneous phage production. The color of branches and strain labels indicate species as identified by ITS region and 16S rDNA DNA sequencing, respectively: *B. japonicum* (blue), *B. elkanii* (red), *B. diazoefficiens* (green), and *B. ottawaense* (purple). The outgroup is *Caulobacter crescentus* (black). Spontaneous phage production levels are indicated by the color of the outer bars: Not determined (black), Not Detectable (no color), Low (teal), Medium (dark green), High (yellow), and Very High (rose) VLP categories. Strains S02B, S06A, S06B, S12J, and S12K possess multiple ITS regions (denoted by numerical suffixes) that were cloned and sequenced by Joglekar et al. [3].

Phylogenetic analysis of the ITS region confirmed that phage production was not dependent on species or phylogenetic placement (Figure 2). Strains belonging to the same clades within species had different levels of observed VLP production. For instance, *B. japonicum* strains S06E-Bj and S07H-Bj, both isolated from Sussex County farms, had ITS regions of 99.88% sequence identity. However, S06E-Bj spontaneously produced a Very High number of VLPs, while S07H-Bj did not produce detectable VLPs. Similarly, *B. diazoefficiens* strains K06L-Bd and K07G-Bd, both isolated from Kent County farms, shared a common ITS clade and produced Very High and Not Detectable levels of phages, respectively.

### 3.2. Morphological Characteristics of Lysogenic Phages

**Figure 3:**
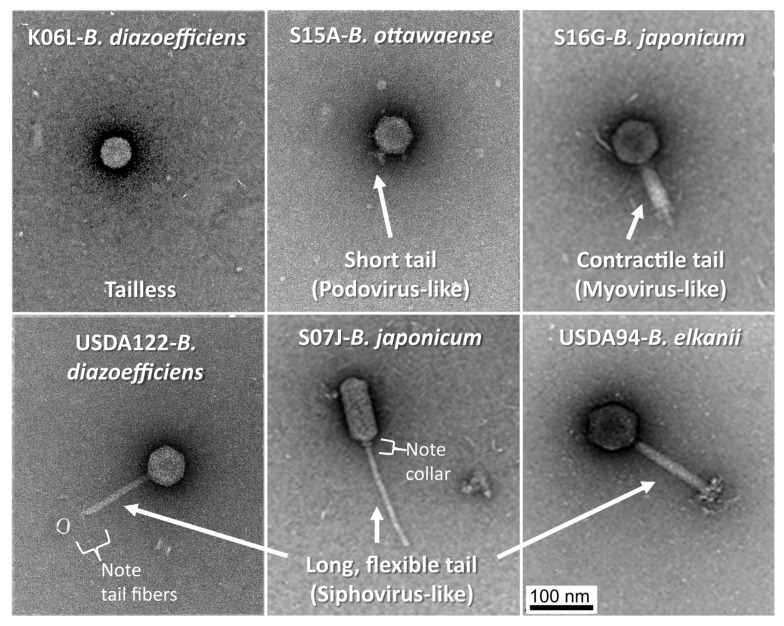
Representative morphotypes of phages produced spontaneously by six strains of soybean *Bradyrhizobium* species in laboratory culture. Phages were negatively stained (2% uranyl acetate) and observed using transmission electron microscopy. All images are at the same scale.

**Figure 4:**
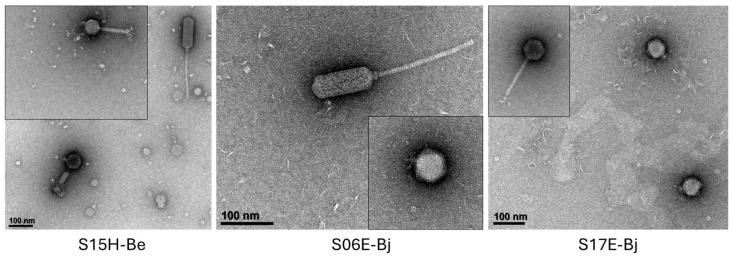
Representative virions produced spontaneously by three polylysogenic strains of soybean *Bradyrhizobium*: *B. elkanii* S15H, *B. japonicum* S06E, and *B. japonicum* S17E. For a given strain (panel), all sub-images are at the same scale.

Morphotypes of temperate phages from 44 hosts spontaneously producing greater than 2.0 × 10^8^ VLP mL^−1^ were documented using TEM. This included phages from 21 *B. japonicum*, 14 *B. diazoefficiens*, 7 *B. elkanii*, and 2 *B. ottawaense* strains. Eleven of the 44 strains (25.0%) produced tailed virions (letter code T in Figure 1, Figure S1), while an additional 15 strains (34.1%) produced apparently tailless virions, having capsids that were clearly icosahedral (letter code N in Figure 1) (Figure 1, Figure 3 and Figure 4). A third category representative of 13 strains (29.5%) yielded particles having questionable icosahedral morphology (letter code Q in Figure 1). Examination of TEM images could not confirm virion production for five of the 44 strains examined (11.4%; letter code U in Figure 1). However, particles having morphologies similar to extracellular vesicles (EVs) (Figure S2) were observed for 38 of the 44 strains (86.4%) and for all five of the strains that did not produce definite or questionable virions; it is possible that some of these contained DNA [35] and contributed to the VLP counts (Figure 1). It should also be noted that separate cultures were used for VLP counts and TEM imaging; as such, it is possible that virions were not produced in the latter cultures for unknown reasons by the five strains for which virions were not observed by TEM imaging.

TEM revealed diverse phage morphologies among the 587 virions observed (Figure 3). At least one *B. elkanii* strain (S15H-Be) and two *B. japonicum* strains (S06E-Bj and S17E-Bj) were polylysogens, spontaneously producing viruses of more than one morphotype (letter code P in Figure 1 and Figure 4). Strain S15H-Be produced three morphologically distinct phages, specifically two siphovirus-like phages, one with prolate (capsule-shaped) capsids and a second with icosahedral capsids, as well as myovirus-like phages. Both S06E-Bj and S17E-Bj produced podovirus-like and siphovirus-like phages, the latter phages possessing two distinct capsid (prolate and icosahedral, respectively) and tail morphologies. The phages spontaneously produced by the apparently non-polylysogenic *Bradyrhizobium* strains examined using TEM also exhibited considerable morphologic diversity.

**Figure 5:**
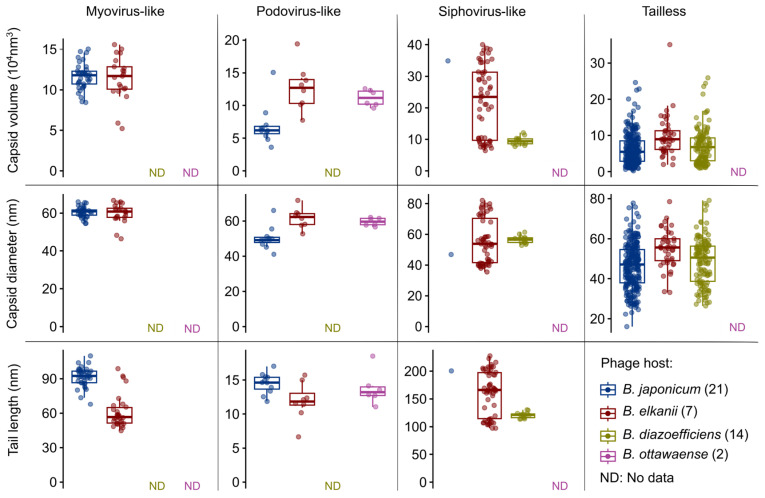
Distribution of morphological measurements for phages produced spontaneously by soybean *Bradyrhizobium* species in laboratory culture. Measurements of capsid diameter and tail length and calculated capsid volumes for all virions (n = 587) were obtained from 44 bradyrhizobia cultures examined using transmission electron microscopy. Phages are grouped by morphotype (columns). Morphotypes are further segregated by *Bradyrhizobium* host species: *B. japonicum* (blue), *B. elkanii* (red), *B. diazoefficiens* (green), and *B. ottawaense* (purple). ND signifies no data (phage morphotype was not observed). The tailless category includes virions possessing clearly icosahedral and questionable icosahedral capsid morphologies.

A majority (71%) of virions possessing clearly icosahedral or questionable icosahedral morphologies imaged across all species were apparently tailless and exhibited a wide range of capsid diameters of approximately 20 to 80 nm. Capsids of myovirus-like phages observed for *B. japonicum* and *B. elkanii* were of similar size (mean capsid volume ~5.7 × 10^4^ nm^3^), but associated tail lengths varied: 91.56 ± 8.3 nm and 61.06 ± 14.14 nm, respectively (Figure 5). The morphologies of podovirus-like phage produced by *B. ottawaense*, *B. japonicum*, and *B. elkanii* varied between species. Phages in this morphotype produced by *B. japonicum* possessed smaller capsid diameters than the other two species and longer tails than *B. elkanii* and possibly also *B. otttawaense*. Siphovirus-like phages produced by *B. elkanii* exhibited a large range of capsid diameters (33 to 82 nm) and tail lengths (97 to 227 nm) and appeared to segregate into three subgroups.

## 4. Discussion

A remarkable 69% of UDBCC strains examined exhibited significant production of nucleic acid-positive virus-sized particles (VLPs), i.e., in the absence of any apparent external inducing agents, at levels ranging from 10^7^ to over 10^9^ VLP mL^−1^ of culture. Both VLP-producing and non-producing cultures grew normally, with no visual evidence of lysis occurring in the former. Of the 44 strains observed by TEM, 26 (59.1%) produced morphologically identified phages, while an additional 13 (29.5%) produced particles of questionable icosahedral morphology. Possibly contributing to the VLP counts (Figure 1), 38 (86.5%) of the strains produced particles resembling EVs, some of which may have contained DNA [35]. Similar high levels of spontaneous phage production have been reported by others for a limited number of genera (e.g., [7,10,36]). Nanda et al. [11] summarized various mechanisms of spontaneous induction, including stochastic events resulting from spontaneous cell-to-cell fluctuations in the levels of induction repressor molecules. With respect to the current study, we speculate that a subpopulation of cells enters a lytic cycle while the remaining cells continue to grow normally. Alternatively, it is conceivable that some of the tailless phages are produced chronically without causing lysis in a manner similar to that reported by Liu et al. [37], although the tailless phages observed in their study possessed lipid envelopes, which were not apparent in our study. Because our VLP counts were taken during the late-log to early-stationary phase of bacterial growth, the spontaneous phage production observed may be due to a cell stress response from nutrient depletion [8], quorum sensing events [14,15], or other unidentified biotic or abiotic stimuli such as the production of secondary metabolites [13]. However, in opposition to the cellular stress argument, Joglekar et al. [4] reported parallel increases in bacterial and VLP numbers for two of the four *Bradyrhizobium* strains examined in their study, i.e., VLPs were continually produced throughout the duration of host growth. Time-course monitoring of phage production and culture conditions during bacterial growth and the use of additional or modified growth media may provide insight regarding induction stimuli.

All four species within the subset of UDBCC strains produced detectable VLPs in culture. Production was not specific to ITS region identity (Figure 2) or phenotypic groupings based on serological reaction or cellular fatty acid composition, as described by Joglekar et al. [3]. Moreover, of the 29 Delaware farms represented in the UDBCC subset, at least one culture obtained from 27 farms produced detectable VLPs. Twelve of these latter farms also yielded root nodule isolates that did not produce detectable VLPs. Similarly, six of the 13 USDA reference strains examined, representing geographically diverse regions of the U.S. distinct from Delaware, also produced detectable VLPs.

Given the frequency and magnitude of spontaneous phage production observed, it is reasonable to speculate that this cellular process is an important driver of genetic and phenotypic diversity among soybean *Bradyrhizobium*. The evidence of specific transduction by spontaneously produced phages reported by Joglekar et al. [4] supports this supposition. High incidences of lysogens may suggest the ecological and evolutionary significance of lysogeny for bacteria in the soil [17] and other environments [11]. It is well established that traits conferred by resident prophages can enhance host fitness, such as better resistance to antibiotics and osmotic stress, faster host growth rates, and increased biofilm formation due to the release of eDNA [11,38]. Spontaneous phage production may also increase prophage fitness via infection and lysis of non-lysogenic bacterial competitors, thereby giving the lysogenized host a competitive advantage. Alternatively, released phages could promote the spread of their genes in a community via the lysogenic conversion of non-lysogens. Root nodules represent a carbon-rich environment with an active and numerically dense population of (brady)rhizobia and sometimes other bacterial taxa (e.g., [39]) that may provide an ideal environment for viral infection.

Transmission electron microscopy revealed diverse morphological features among phages recovered from culture supernatants of 44 bradyrhizobia strains producing over 2 × 10^8^ VLP mL^−1^. Phages with icosahedral capsids and long, flexible, non-contractile tails (siphovirus-like) were similar to previously recorded lytic phages infecting alfalfa *Rhizobium melilotii* [40], groundnut *Bradyrhizobium* [41], and soybean *B. japonicum* [42]. Phage capsid diameters observed in the three studies ranged from 55 to 80 nm, compared with average capsid diameters of 40 to 73 nm in the current study. Appunu and Dhar [42] additionally reported a short-tailed (podovirus-like) *B. japonicum* lytic phage having a 65 nm capsid diameter and 25 nm tail length; corresponding mean values in our study ranged from 49 to 66 nm and 12 to 15 nm, respectively.

Interestingly, 71% of the virions observed using TEM were apparently tailless. Brum et al. [43] discovered that tailless icosahedral viruses dominated (average 66 to 85%) in several oceanographic regions. Similar to our observations, Alexeema et al. [7] reported high levels of spontaneously produced apparently tailless phages (>10^8^ mL^−1^) by *Lactococcus lactis* in routine laboratory culture. It is possible that the phage tails in our study were removed during the filtering and concentration steps used in preparing virions for TEM characterization, leading to misclassification. A high occurrence of tailless viruses in marine and soil environments was attributed to tail loss during similar sample preparation [44,45]. However, it is worth noting that virion TEM images for only three strains in our study revealed the presence of detached tails (Figure S1), suggesting both that tail detachment did not commonly occur during processing and that any tails that were detached were concentrated with intact virions and observable in the TEM images. It is possible that some or all of the apparently tailless virions observed were defective or incomplete. It is also possible that many of the supposed tailless virions we observed possessed inconspicuous or positionally obscured tails that were not apparent in the TEM images. The possible presence of tail structural genes within the genomes of the apparently tailless phages may help support this hypothesis, as well as the possibility that these tailless phages are a result of defective assembly. Lastly, it is also possible that strains producing high VLP counts (i.e., the 44 strains examined using TEM), but for which we were unable to confirm the presence of virions by TEM (n = 5), produced extracellular vesicles containing DNA [35]. For those strains that did produce identifiable virions, the putative EVs observed may have resulted from phage-mediated explosive cell lysis [46]. Procedures for unambiguously determining EV production were beyond the scope of this study. Nevertheless, EVs produced by bradyrhizobia may serve any number of purposes such as gene transfer [47] or as phage decoys [48].

Lysogenized bacteria are often resistant to secondary infection [49], although there are many reports of polylysogeny in the literature (e.g., [50,51,52,53]). We observed clear evidence of polylysogeny for three of the 44 spontaneously producing strains examined using TEM. However, it is likely that more accessions in the UDBCC would prove to be polylysogens if chemical or other inducing agents were applied to cultures. Increasing the number of virions imaged per strain might reveal additional spontaneously produced morphotypes occurring at lower abundances. For example, Joglekar et al. [4] previously reported two (pro)phages in USDA76-Be, whose virions were produced at different abundances and were morphologically and genetically distinct, although we observed only one morphotype in the present study.

In conclusion, this study revealed a high frequency of abundant spontaneous phage production and possibly production of DNA-containing EVs within a diverse collection of soybean *Bradyrhizobium*. Furthermore, a wide range of phage morphotypes was observed, suggesting a correspondingly high level of genetic diversity. Although the mechanism(s) of spontaneous phage production in the current study is presently unknown, the high incidence and numbers of phages observed support the Joglekar et al. [4] hypothesis that infection and transduction events promoted by spontaneous production are an important component of the soybean *Bradyrhizobium* mobilome and drivers of evolution in the genus.

## Data Availability

The raw data supporting the conclusions of this article will be made available by the authors on request.

## Supplementary Materials

The following supporting information can be downloaded at: https://www.mdpi.com/article/10.3390/v16111750/s1. Figure S1: Intact virions and detached tails of phages spontaneously produced by soybean *Bradyrhizobium* strains S15H-Be, S17E-Bj, and USDA94-Be. Detached tails were not observed for other host strains examined, Figure S2: Representative putative extracellular vesicles produced by various strains of soybean *Bradyrhizobium* spp. The scale bar in all cases represents 100 nm.
