# Supplementary material for: Soybean Bradyrhizobium spp. Spontaneously Produce Abundant and Diverse Temperate Phages in Culture
Source: Viruses. 2024 Nov 7;16(11):1750. doi: 10.3390/v16111750 (PMC11599138; doi:10.3390/v16111750)
Supplement: Supplementary file 1 [file viruses-16-01750-s001.zip › Figure S1.pdf]

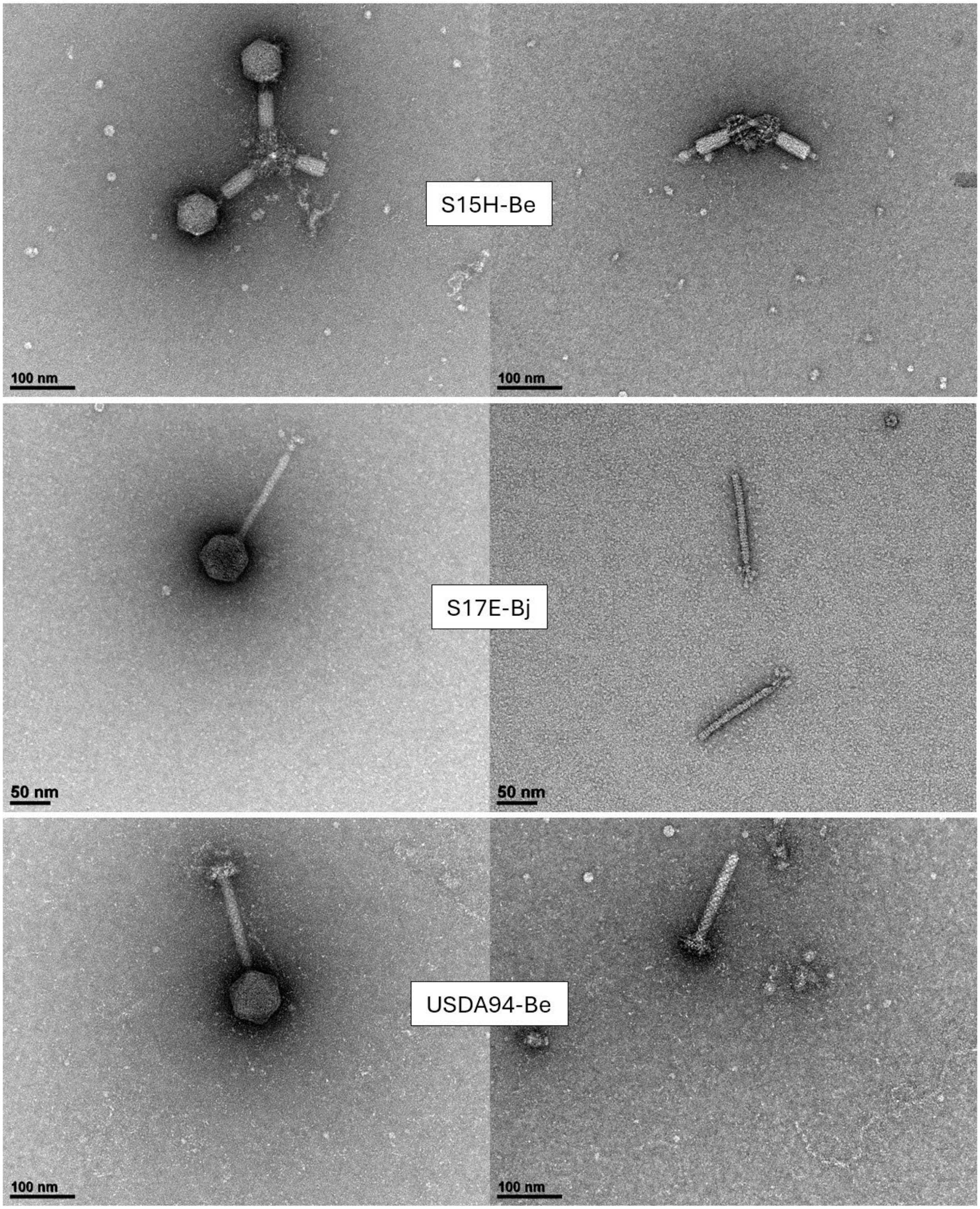

Figure S1: Intact virions and detached tails of phages spontaneously produced by soybean *Bradyrhizobium* strains S15H-Be, S17E-Bj, and USDA94-Be. Detached tails were not observed for other host strains examined.
