# Supplementary material for: Soybean Bradyrhizobium spp. Spontaneously Produce Abundant and Diverse Temperate Phages in Culture
Source: Viruses. 2024 Nov 7;16(11):1750. doi: 10.3390/v16111750 (PMC11599138; doi:10.3390/v16111750)
Supplement: Supplementary file 1 [file viruses-16-01750-s001.zip › Figure S2.pdf]

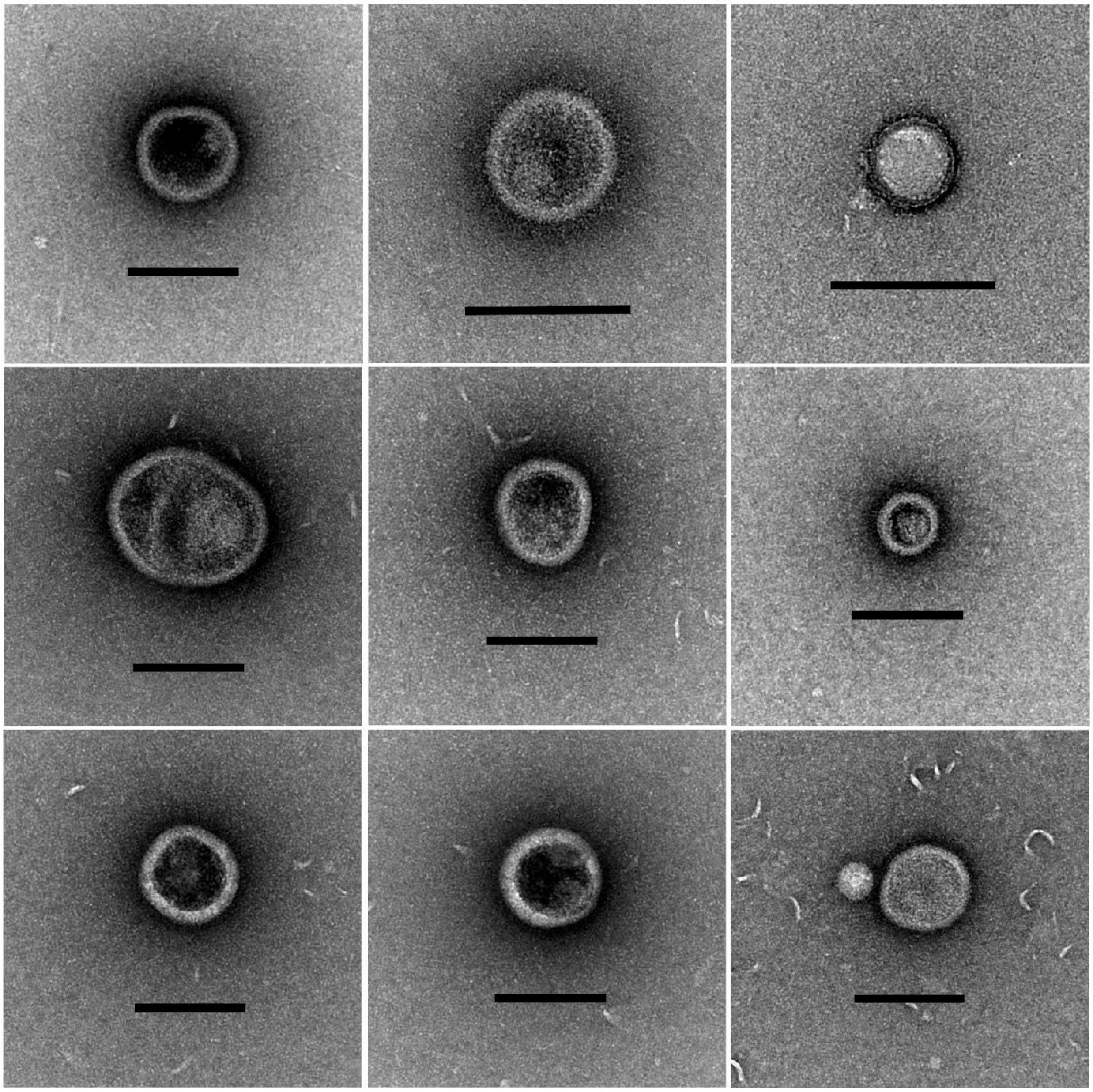

Figure S2: Representative putative extracellular vesicles produced by various strains of soybean *Bradyrhizobium* spp. The scale bar in all cases represents 100 nm.
